# A Predictive Nomogram for Red Blood Cell Transfusion in Pheochromocytoma Surgery: A Study on Improving the Preoperative Management of Pheochromocytoma

**DOI:** 10.3389/fendo.2021.647610

**Published:** 2021-03-11

**Authors:** Ying Guo, Lili You, Huijun Hu, Anli Tong, Xiaoyun Zhang, Li Yan, Shaoling Zhang

**Affiliations:** ^1^ Department of Endocrinology, Sun Yat-sen Memorial Hospital, Sun Yat-sen University, Guangzhou, China; ^2^ Department of Radiology, Sun Yat-sen Memorial Hospital, Sun Yat-sen University, Guangzhou, China; ^3^ Department of Endocrinology, Peking Union Medical College Hospital, Beijing, China

**Keywords:** pheochromocytoma, blood transfusion, nomogram, prediction model, surgery

## Abstract

**Purpose:**

Surgery is the major treatment option for pheochromocytoma but carries potential risks, including hemorrhage and hemodynamic instability. Even with laparoscopic adrenalectomy, intraoperative blood transfusion happens from time to time, but few studies have investigated risk factors. For the first time we develop and validate a nomogram for prediction of red blood cell transfusion in pheochromocytoma surgery.

**Methods:**

There were 246 patients in our center and 56 patients in Peking Union Medical College Hospital, who underwent pheochromocytoma surgery, enrolled in the study. We incorporated clinical and radiological risk factors, and presented this with a nomogram. Lasso regression model was used for feature selection. Logistic regression analysis was performed to identify the odd ratios. The performance of the nomogram was assessed with respect to its discrimination, calibration and clinical usefulness.

**Results:**

Thirty-two features were reduced to five, which were phenoxybenzamine use, phenoxybenzamine treatment duration, preinduction heart rate, tumor diameter and surgical procedure. The model showed good discrimination (C-index, 0.857; 95% CI, 0.781–0.836) and application in the validation sets also gave good discrimination (internal validation: C-index, 0.831; 95% CI, 0.750–0.822; external validation: C-index, 0.924; 95% CI, 0.766–1.000). Calibration tested with the Hosmer-Lemeshow test yielded a good agreement between prediction and observation (training P=0.358; internal validation P=0.205; external validation P=0.395). Odd ratios of phenoxybenzamine use, phenoxybenzamine treatment duration, preinduction HR, tumor diameter and open surgery were 13.32 (95% CI, 1.48–197.38; P = 0.034), 1.04 (95% CI, 0.99–1.08; P = 0.092), 1.04 (95% CI, 1.01–1.08; P=0.006), 1.03 (95% CI, 1.02–1.06; P<0.001), 17.13 (95% CI, 5.18–78.79; P<0.001), respectively. Decision curve analysis demonstrated the clinical usefulness of the nomogram.

**Conclusions:**

This study presents a nomogram that may be used to facilitate the prediction of red blood cell transfusion in pheochromocytoma surgery and help to do the preoperative management more efficiently.

## Introduction

Pheochromocytoma (PCC) is a catecholamine secreting tumor arising from chromaffin cells of the adrenal medulla. Circulating catecholamines cause a series of clinical symptoms such as elevated blood pressure, palpitation, headache, as well as heart, brain, kidney and other organ complications (1).

Surgery is the major treatment option but carries potential risks, including hemorrhage and hemodynamic instability, due to the tumoral release of catecholamines during anesthetic induction and tumor manipulation. The mortality associated with surgical resection has significantly improved from 20–45% to 2.9% (2), largely due to the wide use of adrenergic blockade and advances in surgical and anesthetic practice during the past three decades (3). However, surgical resection of PCC still poses significant clinical management challenges. Preoperative management is the key to safe surgery (4, 5).

There is currently no standardized method to assess operative risk for PCC surgery. Based on the available literature, clinicians take age, tumor size, preoperative blood pressure (BP), orthostatic hypotension, body weight change, hematocrit (HCT) and heart rate (HR) as the predictors of hemodynamic stability (6, 7), which is crucial for intraoperative safety. Meanwhile, due to the rich vascularity of PCC, even with laparoscopic adrenalectomy, hemorrhage stays a challenge to surgeons. Therefore intraoperative blood transfusion still happens from time to time, and the global blood product shortage increases the difficulty of such operations to a certain extent. But few studies have investigated risk factors of blood transfusion during PCC surgery to date. Here, we address the question: Is there a way to predict the intraoperative red blood cell (RBC) transfusion before surgery? To answer the question, we develop and validate a nomogram that incorporated both the radiological and clinical risk factors, which may give us some ideas about an individualized prediction of intraoperative RBC transfusion in PCC surgery and help to do the preoperative management more efficiently.

## Materials and Methods

### Ethics Statement

This study was approved by the Institutional Review Board at Sun Yat-sen Memorial Hospital, Sun Yat-sen University. All the data were collected and analyzed after obtaining informed consent by participants.

### Subjects

Patients who underwent surgery for PCC, of which the diagnoses were confirmed by postoperative pathological examination (8), from January 2000 to October 2020 in Sun Yat-sen Memorial Hospital, Sun Yat-sen University and from January 2018 to October 2020 in Peking Union Medical College Hospital, were enrolled. Patients from Peking Union Medical College Hospital served as the external validation group. Patients who met any of the following criteria were excluded: (1) diagnosed metastatic PCCs; (2) extensive resection involving adjacent non-tumor organs; (3) surgery for recurrent or bilateral PCCs; (4) with incomplete medical records.

### Surgical Quality Control

All surgeries were conducted under general anesthesia. Furthermore, all operations were performed by the same surgical team with experience in the treatment of PCC. Two chief surgeons were involved in the surgery.

### Data Collection

We collected demographic and preoperative clinical data, pharmacological and family history, hemodynamics, biochemical and radiographic results, operation and anesthesia records. Catecholamines and their metabolites in plasma and urine were measured by radioimmunoassay methods (ALPCO, Salem, NH, US). Blood parameters were measured using a Sysmex automated blood cell counter (Sysmex XE-2100). Plasma albumin and glucose level were measured using a Mindray automatic biochemical analyzer (Mindray BC-31s). Pathoglycemia, including impaired fasting glucose (IFG), impaired glucose tolerance (IGT) and diabetes, was defined according to American Diabetes Association (ADA) guidelines updated in 2020 (9). Tumor size was derived from the maximum measurement in millimeter (mm). From 72 h before the operation, preoperative systolic blood pressure (SBP) and diastolic blood pressure (DBP) were measured and recorded every 6 h. The BP and HR were measured while seated, having the patient sit quietly for more than 15 min before measurement and avoid caffeine, exercise, eating and smoking for at least 60 min before measurement. The BP fluctuation was defined as the maximum BP minus the minimum BP. The preinduction BP and HR were defined as the BP and HR value measured in the morning of the surgery.

### Preoperative Preparation

A non-selective adrenergic blockade, phenoxybenzamine, was started before surgery to control BP and was titrated according to BP level and tolerability. A beta-adrenoceptor blockade was added in patients with tachycardia after administration of phenoxybenzamine. A calcium channel blockade (CCB) was added if the use of phenoxybenzamine in a sufficient/tolerable dose did not achieve normotension. A high-sodium diet and fluid intake were encouraged to reverse catecholamine-induced blood volume contraction (8).

### Statistics

Statistical analyses were performed using RStudio software. All tests were two-tailed, and a two-sided value of P<0.05 was considered statistically significant. Continuous variables were presented as the means ± standard deviation for normally distributed data and Student’s t-test was used to compare the differences in characteristics of the patients, whereas variables with a skewed distribution were presented as the median (interquartile range, IQR) and differences between groups were tested with Mann-Whitney U test. For the categorical variables, the data were presented as frequencies (percentage) and was compared using the Chi-square test. The least absolute shrinkage and selection operator (LASSO) regression analysis, a method that could minimize the impact of human factors on feature selection, was used to identify the important features associated with intraoperative RBC transfusion in the training set. The glmnet package in R was used for LASSO regression analysis. Odd ratios (ORs), 95% confidence interval (CI) and probability values were calculated by logistic regression analysis.

To evaluate the predictive abilities of this model, an index of the probability of concordance (C-index) was calculated among predicted and actual outcomes. The area under the curve (AUC) in receiver operating characteristic (ROC) analysis was used to evaluate the predictive accuracy of RBC transfusion. Hosmer-Lemeshow tests were sued to assess the calibration of the nomogram. Decision curve analysis (DCA) was performed to assess the clinical usefulness of nomogram by evaluating net benefits at various threshold probabilities in training and validation sets.

## Results

### Characteristics of the Subjects

A total of 246 patients from our center and 56 patients from Peking Union Medical College Hospital were finally included in the study. We randomly split the patients from our center into training (189 subjects, 76.83%) and internal validation (57 subjects, 23.17%) subsets in a 7:3 ratio using simple random sampling method. There was no significant difference in the demographic, clinical, biochemical or radiological parameters between the training and internal validation sets (see **Supplementary Table 1**, which shows characteristics of patients underwent PCC surgery).

The median age was 46.50 (33.25, 56.00) years old. Hypertension was the most common comorbidity (201 cases, 81.71%). The prevalence of clinical symptoms, such as headache, palpitation and sweating was 71.14%. There were 51.63% cases suffered from diabetes, IFG or IGT during disease. The mean diameter of tumor was 52.06 ± 26.31 mm. Elevated catecholamines/metabolites levels were found in 205 (83.33%) cases.

Non-selective adrenergic blockade, phenoxybenzamine, was commonly used as the preoperative preparation, while 15 patients did not have preoperative adrenergic blockade treatment. Phenoxybenzamine treatment duration was 18.46 ± 11.79 days. 112 patients (45.53%) were given beta-adrenoceptor blockades, while 57 patients (23.17%) were given CCBs. The preinduction heart rate was 80 (76, 90) bpm, which was still higher than the level recommended by current guidelines (8, 10).

The majority of patients (183 cases, 74.39%) were American Society of Anesthesiologists (ASA) physical status III. There were 201 patients (81.71%) underwent laparoscopic adrenalectomy. RBC transfusion was performed in 73 cases (29.67%), of which 41 underwent laparoscopic adrenalectomy and 32 underwent open surgery (**Table 1**).

**Table 1:** Comparison between RBC transfusion and non-RBC transfusion groups.

Variables	Training data set (n = 189)			Validation data set (n = 57)		
	Transfusion (n = 61)	Non-transfusion (n = 128)	P-value	Transfusion (n = 12)	Non-transfusion (n = 45)	P-value
Male	27 (44.26%)	59 (46.09%)	0.936	8 (66.67%)	19 (42.22%)	0.237
Age, years	43.0 (33.0,52.0)	49.0 (33.0, 57.5)	0.131	44.0 (39.0, 57.5)	50.0 (37.0, 56.0)	0.652
Family history	3 (4.92%)	3 (2.34%)	0.345	1 (8.33%)	2 (4.44%)	0.529
Hypertension	51 (83.61%)	103 (80.47%)	0.604	11 (91.67%)	36 (80.00%)	0.345
Elevated catecholamines	53 (86.89%)	107 (83.59%)	0.821	11 (91.67%)	34 (75.56%)	0.224
Tumor diameter, mm	67.53 ± 32.39	44.60 ± 18.00	<0.001	69.67 ± 38.89	47.57 ± 22.63	0.082
PBZ use	59 (96.72%)	119 (92.97%)	0.303	12 (100%)	41 (91.11%)	0.284
PBZ duration, day	20.36 ± 8.64	17.09 ± 10.33	0.024	16.00 ± 3.52	20.44 ± 18.58	0.138
Preoperative SBP fluctuation, mm Hg	35.93 ± 16.72	30.96 ± 13.26	0.044	33.83 ± 19.31	29.04 ± 12.65	0.43
Preoperative DBP fluctuation, mm Hg	22.03 ± 10.10	20.87 ± 8.41	0.436	21.75 ± 11.41	21.07 ± 10.19	0.853
Preinduction SBP, mm Hg	130.00 (118.00, 142.00)	132.00 (115.00, 141.00)	0.786	126.00 (118.00, 146.25)	127.00 (115.00, 141.00)	0.695
Preinduction DBP, mm Hg	80.00 (72.00, 92.00)	77.50 (70.00, 87.00)	0.190	80.00 (76.00, 87.75)	79.00 (72.00, 87.00)	0.557
Preinduction HR, bpm	88.00 (80.00, 93.00)	80.00 (73.00, 88.00)	<0.001	90.50 (81.50, 96.00)	80.00 (74.00, 84.00)	0.011
Laparoscope adrenalectomy	35 (57.38%)	120 (93.75%)	<0.001	6 (50%)	40 (88.89%)	0.002
Open Surgery	26 (42.62%)	8 (6.25%)		6 (50%)	5 (11.11%)	

RBC, red blood cell; PBZ, phenoxybenzamine; SBP, systolic blood pressure; DBP, diastolic blood pressure; HR, heart rate.

Compared with non-transfusion group, patients in transfusion group had larger tumor volume, longer preoperative use of phenoxybenzamine, larger preoperative SBP fluctuation and faster preinduction HR, but there was no difference in gender, age, family history, prevalence of hypertension, additional medical history and medications, incidence of positive symptom, BMI, preoperative DBP fluctuation, preinduction BP or biochemical parameters between the two groups (**Table 1** and **Supplementary Table 2**). Meanwhile, more patients in transfusion group were performed open surgery (42.62% vs. 6.25%, as shown in **Table 1**).

### Feature Selection and Risk Factor Analysis of Intraoperative RBC Transfusion

**Figure 1:**
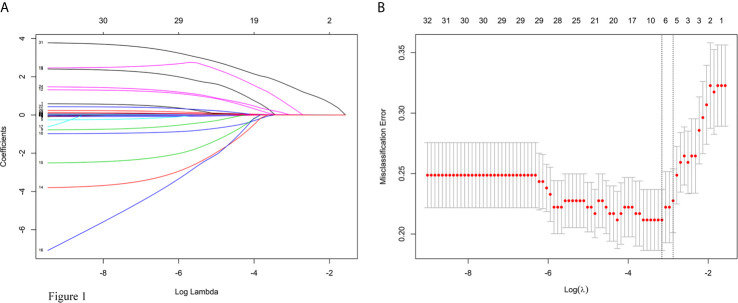
Texture feature selection using the least absolute shrinkage and selection operator (LASSO) logistic regression model. **(A)** LASSO coefficient profiles of the 32 texture features. **(B)** Tuning parameter (lambda) selection in the LASSO model used 10-fold cross-validation *via* minimum criteria for risk of intraoperative RBC transfusion.

A λ value of 0.056 with log (λ), −2.878, was chosen (1 standard deviation) by LASSO regression analysis according to 10-fold cross-validation based on the minimum criteria. Features with non-zero coefficients were selected as the risk factors of intraoperative RBC transfusion (**Figures 1A, B**), while features excluded by LASSO analysis were more inclined to no difference between groups. Thirty-two texture features (see **Supplementary Table 3** for all contents) were reduced to five (**Figures 1A, B**), which were phenoxybenzamine use, phenoxybenzamine treatment duration, preinduction HR, tumor diameter and surgical procedure (**Table 2**).

**Table 2:** Risk factor analysis of intraoperative RBC transfusion.

Intercept and variable	Model in training dataset		
	β	OR(95%CI)	P-value
Intercept	−9.918		<0.001
PBZ Use	2.590	13.32 (1.48–197.38)	0.034
PBZ Treatment Duration	0.035	1.04 (0.99–1.08)	0.092
Preinduction HR	0.044	1.04 (1.01–1.08)	0.006
Tumor Diameter	0.034	1.03 (1.02–1.06)	<0.001
Surgical Procedure (Open vs. Laparoscope)	2.841	17.13 (5.18–78.79)	<0.001

β is the regression coefficient.

RBC, red blood cell; OR, odd ratio; PBZ, phenoxybenzamine; HR, heart rate.

Multivariate logistic regression analysis was also performed to identify the OR values as shown in **Table 2** (OR values of phenoxybenzamine use, phenoxybenzamine treatment duration, preinduction HR, tumor diameter and open surgery were 13.32 (95% CI, 1.48–197.38; P = 0.034), 1.04 (95% CI, 0.99–1.08; P = 0.092), 1.04 (95% CI, 1.01–1.08; P = 0.006), 1.03 (95% CI, 1.02–1.06; P < 0.001), 17.13 (95% CI, 5.18–78.79; P < 0.001), respectively.

### Development and Validation of an Individualized Prediction Model for RBC Transfusion

**Figure 2:**
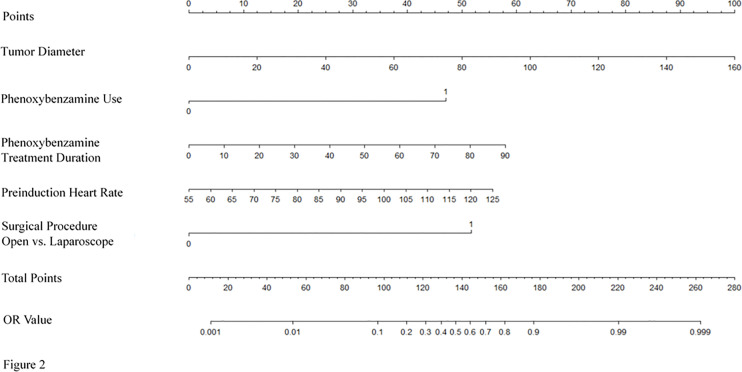
Nomogram for predicting intraoperative RBC transfusion. The nomogram was developed in the training set, with the phenoxybenzamine use, phenoxybenzamine treatment duration, preinduction heart rate, tumor diameter and surgical procedure incorporated.

**Figure 3:**
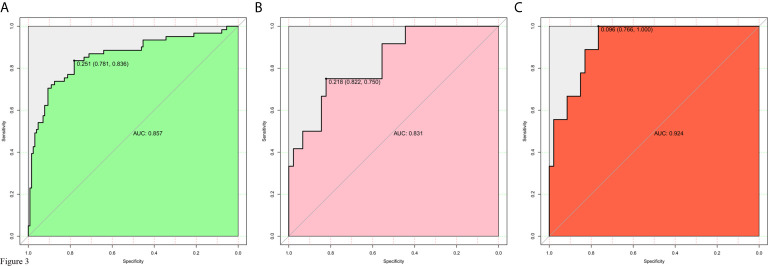
Receiver operating characteristic (ROC) curves for evaluating the nomogram model’s discrimination performance in both training and validation sets. **(A)** ROC curve of the nomogram in the training set. **(B)** ROC curve of the nomogram in the internal validation set. **(C)** ROC curve of the nomogram in the external validation set. The AUC of ROC curves plot sensitivity against 1-specificity of the nomogram.

The model for individualized prediction of intraoperative RBC transfusion, which incorporated phenoxybenzamine use, phenoxybenzamine treatment duration, preinduction HR, tumor diameter and surgical procedure, was presented as a nomogram (**Figure 2**). We constructed ROC curves to evaluate the predictive ability of the nomogram model to predict the prevalence of intraoperative RBC transfusion in both training set (**Figure 3A** **)** and internal validation set (**Figure 3B**). The AUC of ROC curve in training and internal validation were 0.857 (95% CI, 0.781–0.836) and 0.831 (95% CI, 0.750–0.822), respectively. Besides, external validation was conducted (see **Supplementary Table 4**) and the AUC of ROC curve was 0.924 (95% CI, 0.766–1.000) (**Figure 3C**). The calibration of the nomogram demonstrated good agreement between prediction and observation, while the Hosmer-Lemeshow test yielded a nonsignificant statistic in training (P=0.358), internal validation (P=0.205) and external validation (P=0.395) sets.

### Clinical Use

**Figure 4:**
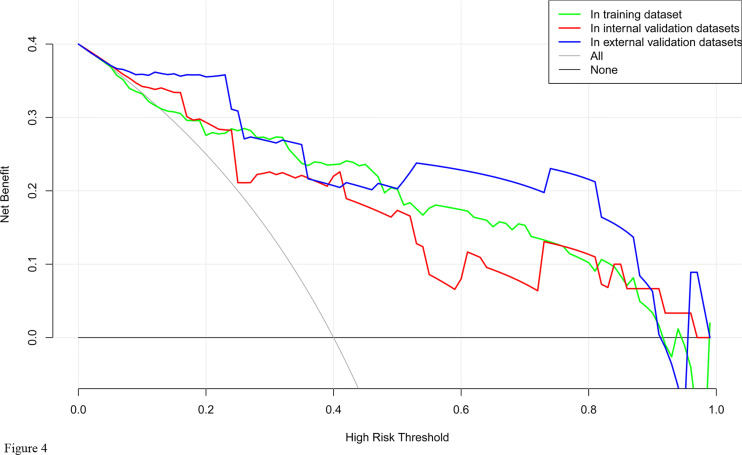
Decision curve analysis for the nomogram model of intraoperative RBC transfusion in training set, internal validation set and external validation set. The y-axis measures the net benefit. The x-axis indicates the threshold probability of the risk of intraoperative RBC transfusion. The green line represents the nomogram in training set, while the red one represents the nomogram in internal validation set and the blue one represents that in external validation set. The grey line represents the assumption that all patients have intraoperative RBC transfusion. Thin black line represents the assumption that no patient has intraoperative RBC transfusion.

DCA for the nomogram was presented in **Figure 4**, which showed that using the nomogram model to predict intraoperative RBC transfusion added more benefit than either the treat-all-patients scheme or the treat-none scheme in both training and validation sets. Within this range, the net benefit was comparable on the basis of the nomogram.

## Discussion

Currently, there is little study on blood transfusion in PCC surgery. For the first time, we developed and validated a clinical- and radiological- based nomogram for the preoperative individualized prediction of RBC transfusion in PCC surgery. The nomogram integrates phenoxybenzamine use, phenoxybenzamine treatment duration, preinduction HR, tumor diameter and surgical procedure with satisfactory discrimination achieved.

For the construction of the nomogram, 32 texture features were reduced to 5 risk factors by examining the predictor-outcome association by shrinking the regression coefficients with the LASSO method. This method surpasses the method of choosing predictors on the basis of the strength of their univariable association with outcome and minimizes the impact of human factors (11, 12).

The data presented here demonstrate that the nomogram applies well to patients undergoing surgery for PCC. As a nomogram constructed based on data from a single institution and validated by other additional institution, this nomogram has broader applicability. But since the data are based on unilateral PCC, this nomogram is not applicable to patients who undergo surgical resection for recurrent or bilateral disease.

In our study, preoperative phenoxybenzamine treatment and a longer duration showed obvious predictive power of increased RBC transfusion. There was no evident reason that could account for this finding, and we speculated that the observed effect might be the impact of phenoxybenzamine on vascular elasticity.

Current guidelines suggest that in order to normalize BP of PCC patients, the preoperative medication should be initiated 7 to 14 days before surgery, and adrenergic blockade is recommended as the first choice mostly (1, 8). Liu et al. (13) reported that preoperative adrenergic blockade treatment duration ≤ 14 days was a risk factor of massive hemorrhage in PCC surgery and increased RBC transfusion rate. Recent studies had provided different viewpoints and questioned the routine preoperative use of adrenergic blockade. Groeben et al. (14, 15) revealed that intraoperative hypotension occurred more with a preoperative adrenergic blockade, which was closely related to the pharmacological effect of phenoxybenzamine on vasodilation. Furthermore, Li et al.’s study (16) found that intraoperative hypotension was an independent risk factor of complications, including RBC transfusion, in patients who underwent PCC surgery. Compared to Liu et al.’s study (13), the average phenoxybenzamine treatment duration in our study was longer than 14 days to meet the control criteria recommended by the guideline (10). Combined with previous studies (14–16), the influence of phenoxybenzamine use on intraoperative RBC transfusion in our study might be due to the decrease of vasoconstriction then affected hemostasis. So far the potential benefit of preoperative adrenergic blockade treatment in controlling preoperative BP and BP fluctuation in PCC surgery remains convinced (7, 17–22). To the authors’ knowledge, there is no randomized controlled trial to determine the ideal application standard of the adrenergic blockade and the existing studies have failed to discuss the influence of treatment duration on perioperative safety. According to our results, the duration of adrenergic blockade treatment is not the longer the better but should be individualized.

Our study observed that faster preinduction HR was associated with more frequent intraoperative RBC transfusion. HR is an important index for evaluating hemodynamics (23, 24) as well as BP and BP fluctuation. A higher preoperative SBP variability in the RBC transfusion group was also proved as shown in **Table 1**. Our results indicated that preoperative hemodynamic instability was closely related to not only intraoperative hemodynamics but also RBC transfusion. Previous studies have proved that preoperative adrenergic blockade treatment served as a major factor affecting the stability of preoperative hemodynamic (7, 17–20), which suggests that hemodynamics might be used as an assessment of the efficacy of preoperative medication in the future.

Our study also showed that larger tumor diameter and open surgery were identified as risk factors of RBC transfusion. In existing studies, tumor size ≥ 5 cm and open surgery were proposed to be independent risk factors for hemorrhage in PCC surgery (13, 25). Yet, studies on the impact of adrenal tumor size on intraoperative hemorrhage have yielded inconsistent results, when assessing adrenal tumors in general (26–30). However, it should be noted that PCCs have a more prominent vascular network than other types of adrenal tumors. Generally, PCCs larger than 5 cm are considered to be at the risk of metastasis (31, 32), and activated angiogenesis is indispensable and critical for establishing metastasis and growth (31, 33). Our study showed a risk of RBC transfusion increased by 3% per unit of tumor diameter (OR, 1.03; 95% CI, 1.02–1.06; **Table 2**). From the current perspective, all PCCs are believed to exhibit malignant potential though only subsets of cases will display full blown malignant properties (34). Our result allowed the possibility that activated angiogenesis was already present even in PCCs smaller than 5 cm. For further growth, activated angiogenesis promoted more vascular network formation. Therefore it was not surprising that larger tumors required more blood vessel manipulations during surgery, which resulted in more bleeding and RBC transfusion. The superiority of the laparoscopic approach over the open approach had been established by numerous studies, including shorter hospital stay, decreased need for intensive care, lower mean operative time and less hemorrhage (35, 36). The collated results of studies showed a mean hemorrhage of 48 to 150 ml in laparoscopic adrenalectomy for PCC while a mean hemorrhage of 164 to 500 ml in open surgery (37). Laparoscopic surgery is the recommended operative approach for most PCC, which can be done *via* a transabdominal or retroperitoneal route, while open resection is only recommended for large (eg, 6 cm) or invasive PCC to ensure complete tumor resection (8). Recently with more and more practices, the size of a tumor is no longer a limitation of laparoscopic surgery (26–28). In a word, laparoscopic surgery can significantly reduce the probability of intraoperative RBC transfusion if possible.

A preoperative estimate of intraoperative RBC transfusion in PCC surgery will help to optimize blood conservation and fully prepare allogeneic blood products to ensure patient safety. Thus the most important and final argument for the usefulness of the nomogram is based on the need to interpret individual needs for additional treatment or care. However, the risk-prediction performance, discrimination and calibration, could not capture the clinical consequences of a particular level of discrimination or degree of miscalibration (12, 38, 39). Therefore, to justify the clinical usefulness, DCA was applied in this study. This method offers insight into clinical consequences on the basis of threshold probability, from which the net benefit could be derived (12, 40, 41). The decision curve showed that using the nomogram in the current study to predict RBC transfusion adds more benefit than either the treat-all-patients scheme or the treat-none scheme.

The limitation of the study is its retrospective design. This study includes patients from a 20-year interval. It is likely that increased experience of the surgical team has impacted RBC transfusion rate over the last 20 years. This effect is hard to quantify and should be considered when interpreting the results of this study. At the same time, methods of catecholamine detection had changed greatly in the past two decades so the effect of catecholamine levels in this study were unable to analyze. Besides, very few patients underwent the surgery without preoperative adrenergic blockade treatment, which could have masked some of the artificial influences. In addition, in our center retroperitoneal laparoscopic adrenalectomy is preferable, it is unknown whether transperitoneal laparoscopic adrenalectomy will give different results (42). On all these counts, further randomized controlled prospective multi-center studies are needed to improve the accuracy of this nomogram.

## Conclusions

In conclusion, our study presents a nomogram that incorporates phenoxybenzamine use, phenoxybenzamine treatment duration, preinduction HR, tumor diameter and surgical procedure, which may be used to facilitate the preoperative individualized prediction of RBC transfusion in PCC surgery and help to do the preoperative management more efficiently. This finding may also support the idea that taking into account factors that span different aspects is the most promising approach to change clinical management.

## Data Availability Statement

The original contributions presented in the study are included in the article/**Supplementary Material**. Further inquiries can be directed to the corresponding author.

## Ethics Statement

The studies involving human participants were reviewed and approved by Institutional Review Board at Sun Yat-sen Memorial Hospital, Sun Yat-sen University. The patients/participants provided their written informed consent to participate in this study.

## Author Contributions

YG, LLY, and SZ conceived and designed the study. YG, HH, AT, and XZ collected and managed the data. YG and LLY analyzed the data and wrote the manuscript. LY and SZ reviewed and edited the manuscript. All authors contributed to the article and approved the submitted version.

## Funding

This work was supported by the Natural Science Foundation of Guangdong Province (2018A030313596) to YG, and the National Natural Science Foundation of China (81970683) and the Natural Science Foundation of Guangdong Province (2020A1515010245) to SZ.

## Conflict of Interest

The authors declare that the research was conducted in the absence of any commercial or financial relationships that could be construed as a potential conflict of interest.
