# Supplementary material for: A Predictive Nomogram for Red Blood Cell Transfusion in Pheochromocytoma Surgery: A Study on Improving the Preoperative Management of Pheochromocytoma
Source: Front Endocrinol (Lausanne). 2021 Mar 11;12:647610. doi: 10.3389/fendo.2021.647610 (PMC8006300; doi:10.3389/fendo.2021.647610)
Supplement: Supplementary file 1 [file Table_1.docx]

| Supplementary Table 1. Characteristics of patients underwent pheochromocytoma surgery | | | | |
| --- | --- | --- | --- | --- |
| Variables | Total  (N=246) | Training dataset  (N=189) | Validation dataset  (N=57) | P-value |
| Male | 113(45.93%) | 86(45.50%) | 27(47.37%) | 0.923 |
| Age, years | 46.5(33.3,56.0) | 46.0(33.0,55.0) | 48.0(38.0,56.0) | 0.348 |
| BMI, kg/m^2^ | 21.85±2.73 | 21.77±2.71 | 22.04±2.79 | 0.487 |
| Tumor Diameter, mm | 52.06±26.31 | 52.00±25.87 | 52.22±27.96 | 0.958 |
| Family History | 9(3.66%) | 6(3.17%) | 3(5.26%) | 0.436 |
| Hypertension | 201(81.71%) | 154(81.48%) | 47(82.46%) | 1.000 |
| Positive Symptom | 175(71.14%) | 135(71.43%) | 40(70.18%) | 0.987 |
| Elevated Catecholamines/Metabolites | 205(83.33%) | 160(84.66%) | 45(78.95%) | 0.275 |
| RBC Count, ^10^12^/L | 4.46(4.05, 4.80) | 4.42(4.04,4.76) | 4.48(4.12,4.97) | 0.547 |
| Hb, g/L | 127(115,138) | 127(115,137) | 131(114,139) | 0.750 |
| HCT | 0.39(0.35,0.41) | 0.38(0.36,0.41) | 0.39(0.35,0.43) | 0.838 |
| Albumin, g/L | 42.2(39.3,44.7) | 42.2(39.1,44.9) | 42.3(39.4,44.5) | 0.959 |
| Pathoglycemia | 127(51.63%) | 103(54.50%) | 24(42.11%) | 0.136 |
| PBZ Use | 231(93.90%) | 178(94.18%) | 53(92.98%) | 0.754 |
| PBZ Treatment Duration, day | 18.46±11.79 | 18.15±9.92 | 19.51±16.64 | 0.559 |
| β-adrenoceptor Blockade Use | 112(45.50%) | 90(47.62%) | 22(38.60%) | 0.295 |
| CCB use | 57(23.17%) | 38(20.11%) | 19(33.33%) | 0.058 |
| Preoperative SBP Fluctuation,mmHg | 31.98±14.53 | 32.57±14.61 | 30.05±14.24 | 0.249 |
| Preoperative DBP Fluctuation,mmHg | 21.24±9.29 | 21.24±8.98 | 21.21±10.36 | 0.983 |
| Preinduction SBP,mmHg | 130(116,142) | 130(116,142) | 127(116,141) | 0.542 |
| Preinduction DBP,mmHg | 79(72,88) | 79(71,89) | 79(73,87) | 0.669 |
| Preinduction HR,bpm | 80(76,90) | 81(76,90) | 80(76,88) | 0.595 |
| ASA score |  |  |  |  |
| II | 51(20.73%) | 38(20.11%) | 13(22.81%) | 0.564 |
| III | 183(74.39) | 143(75.66%) | 40(70.18%) |  |
| IV | 12(4.88%) | 8 (4.23%) | 4(7.02%) |  |
| Surgical Procedure |  |  |  |  |
| Laparoscope | 201(85.37%) | 155(82.01%) | 46(80.70%) | 0.977 |
| Open | 45(14.63%) | 34(17.99%) | 11(19.30%) |  |
| RBC Transfusion | 73(29.67%) | 61(32.28%) | 12(21.05%) | 0.144 |

Abbreviations: BMI, body mass index; RBC, red blood cell; Hb, hemoglobin; HCT, hematocrit; PBZ, phenoxybenzamine; SBP, systolic blood pressure; DBP, diastolic blood pressure; HR, heart rate; CCB, calcium channel blockade; ASA, American Society of Anesthesiologists.

| Supplementary Table 2. Comparison of other analyzed factors between RBC transfusion and non-RBC transfusion groups | | | | | | |
| --- | --- | --- | --- | --- | --- | --- |
| Variables | Training dataset (N=189) | | | Validation dataset (N=57) | | |
|  | Transfusion (N=61) | Non-transfusion (N=128) | P-value | Transfusion (N=12) | Non-transfusion (N=45) | P-value |
| BMI, kg/m^2^ | 22.02±3.04 | 21.70±2.57 | 0.457 | 20.98±2.52 | 22.27±2.78 | 0.151 |
| Positive Symptom | 43(70.49%) | 92(71.88%) | 0.844 | 11(91.67%) | 29(64.44%) | 0.067 |
| Heart Disease | 2(3.28%) | 4(3.13%) | 0.955 | 2(16.67%) | 2(4.44%) | 0.141 |
| Apoplexy | 3(4.92%) | 5(3.91%) | 0.747 | 2(16.67%) | 2(4.44%) | 0.141 |
| CKD | 0(0.00%) | 1(0.78%) | 0.489 | 2(16.67%) | 2(4.44%) | 0.141 |
| COPD | 1(1.64%) | 1(0.78%) | 0.590 | 0(0.00%) | 0(0.00%) | -- |
| RBC Count, ^10^12^/L | 4.57 (4.18, 4.80) | 4.42 (4.00, 4.76) | 0.420 | 4.54 (3.86, 5.08) | 4.46 (4.15, 4.75) | 0.922 |
| Hb, g/L | 126.00 (111.00, 138.00) | 127.00 (117.50, 136.25) | 0.172 | 121.00 (105.75,139.50) | 131.00(118.00,139.00) | 0.439 |
| HCT | 0.38 (0.35, 0.41) | 0.39 (0.36, 0.41) | 0.138 | 0.34 (0.33, 0.43) | 0.39 (0.36, 0.42) | 0.153 |
| Pathoglycemia | 38(62.30%) | 65(50.78%) | 0.137 | 6(50.00%) | 18(40.00%) | 0.533 |
| Plasma Albumin, g/L | 42.30(39.90, 44.55) | 42.05(38.38, 45.15) | 0.907 | 41.66 ± 6.10 | 42.00 ± 3.91 | 0.810 |
| Mean PBZ Daily Dosage, mg/d | 20.00(10.00, 40.00) | 20.00(20.00, 40.00) | 0.175 | 20.00(20.00, 20.00) | 20.00(10.00, 40.00) | 0.920 |
| Total PBZ Dosage, mg | 420.00(250.00, 670.00) | 450.00(232.50, 757.50) | 0.944 | 310.00(250.00, 385.00) | 390.00(212.50, 780.00) | 0.352 |
| β-adrenoceptor Blockade Use | 31(50.82%) | 59(46.09%) | 0.543 | 5(41.67%) | 17(37.78%) | 0.806 |
| β-adrenoceptor Blockade Treatment Duration, day | 22.00(5.00, 61.50) | 13.00(0.00, 47.00) | 0.132 | 52.00(12.25, 68.75) | 13.00(0.00, 33.00) | 0.055 |
| CCB Use | 17(27.87%) | 21(16.41%) | 0.066 | 8(66.67%) | 11(24.44%) | **0.006** |
| CCB Treatment Duration, day | 19.00(0.00, 41.50) | 11.00(0.00, 44.75) | 0.166 | 47.00(20.25, 57.75) | 11.00(0.00, 44.75) | **0.009** |
| ASA Score |  |  |  |  |  |  |
| II | 5(8.20%) | 33(25.78%) | **0.014** | 1(8.33%) | 12(26.67%) | 0.177 |
| III | 54(88.52%) | 89(69.53%) |  | 9(75.00%) | 31(68.89%) |  |
| IV | 2(3.28%) | 6(4.69%) |  | 2(16.67%) | 2(4.44%) |  |

Abbreviations: RBC, red blood cell; BMI, body mass index; CKD, chronic kidney disease; COPD, chronic obstructive pulmonary disease; Hb, hemoglobin; HCT, hematocrit; PBZ, phenoxybenzamine; CCB, calcium channel blockade; ASA, American Society of Anesthesiologist

| Supplementary Table 3. Texture features analysed by LASSO analysis | | | | | |
| --- | --- | --- | --- | --- | --- |
| Demographic Data | Preoperative Clinical Data | Pharmacological History | Preoperative Hemodynamics | Biochemical and Radiographic results | Perioperative Records |
| gender | BMI | PBZ use | preoperative SBP fluctuation | tumor diameter | ASA score |
| age | hypertension | mean PBZ daily dosage | preoperative DBP fluctuation | elevated catecholamines/metabolites | surgical procedure |
| family history | positive symptom | PBZ treatment duration | preinduction SBP | RBC count |  |
|  | pathoglycemia | total PBZ dosage | preinduction DBP | hemoglobin |  |
|  | heart disease | β-adrenoceptor blockade use | preinduction HR | HCT |  |
|  | apoplexy | β-adrenoceptor blockade treatment duration |  | plasma albumin |  |
|  | CKD | CCB use |  |  |  |
|  | COPD | CCB treatment duration |  |  |  |

Abbreviations：BMI, body mass index; SBP, systolic blood pressure; DBP, diastolic blood pressure; HR, heart rate; CKD, chronic kidney disease; COPD, chronic obstructive pulmonary disease; PBZ, phenoxybenzamine; CCB, calcium channel blockade; ASA, American Society of Anesthesiologists; RBC, red blood cell; HCT, hematocrit.

| Supplementary Table 4. Characteristics of external validation group | | | |
| --- | --- | --- | --- |
| Variables | RBC transfusion | Non-transfusion | P value |
| N | 9(16.1%) | 47(83.9%) |  |
| Male | 1(4.2%) | 23(95.8%) | **0.036** |
| Age, years | 44.00±17.45 | 44.15±13.53 | 0.977 |
| Tumor Diameter, mm | 76.67±19.02 | 49.21±19.86 | **<0.001** |
| Phenoxybenzamine Use | 8(88.89%)* | 46(97.87%)* | 0.183 |
| Phenoxybenzamine Treatment Duration, day | 37(29, 51) | 42(27, 57) | 0.315 |
| Preinduction HR,bpm | 85(70, 94) | 80(70, 85) | 0.470 |
| Surgical Procedure |  |  |  |
| Laparoscope | 4(44.44%) | 46(97.87%) | **<0.001** |
| Open | 5(55.56%) | 1(2.13%) |  |

Abbreviations: RBC, red blood cell; HR, heart rate.

*One patient was prescribed with doxazosin.
